# Research progress on the induction of immunogenic cell death in tumor immunotherapy using a sonodynamic therapy nanoparticle delivery system

**DOI:** 10.3389/fimmu.2025.1681773

**Published:** 2025-12-10

**Authors:** Yang Du, Jiangnan Yang, An Xu, Shuai Chen, Deyuan Fu

**Affiliations:** 1 Department of Thyroid and Breast Surgery, Clinical Medical College, Yangzhou University, Yangzhou, China; 2 Department of Basic Medicine, Medical College of Hunan Normal University, Changsha, China; 3 Department of Thyroid and Breast Surgery, Yiyang Central Hospital, Yiyang, China; 4 Department of Thyroid and Breast Surgery, Northern Jiangsu People’s Hospital Affiliated to Yangzhou University, Yangzhou, China

**Keywords:** sonodynamic therapy, immunogenic cell death, drug delivery, nanotechnology, synergistic effect

## Abstract

ICD is critical for enhancing antitumor immune responses in tumor immunotherapy. SDT employs ultrasound to activate Sonosensitizers, generating ROS that induce cytotoxic tumor cell death and trigger ICD through the release of DAMPs. However, standalone SDT faces challenges such as limited Sonosensitizers accumulation and poor tissue specificity. Nanoparticle-mediated SDT addresses these limitations by improving Sonosensitizers delivery, tumor targeting, and biocompatibility. This review explores how nanotechnology enhances SDT to induce ICD, focusing on its integration with chemotherapy and immunotherapy to achieve synergistic antitumor effects. We highlight recent advancements in multifunctional nanoplatforms that optimize ROS production, reprogram the tumor microenvironment, and enhance immune activation. By overcoming the constraints of conventional therapies, nanoparticle-mediated SDT offers a promising strategy for precise, effective, and low-toxicity tumor immunotherapy, with potential for clinical translation.

## Introduction

Although traditional cancer treatments like surgery, chemotherapy, and radiotherapy have been effective in controlling tumor growth, they have significant limitations. These methods often lead to systemic toxicity, drug resistance, and high tumor recurrence rates. For example, surgery often causes damage to normal tissues and is difficult to manage metastatic tumors; chemotherapy may induce multidrug resistance mechanisms, while radiotherapy may damage normal tissues, increasing the risk of secondary cancers (1). Additionally, traditional treatments struggle to address tumor heterogeneity and the complexity of the tumor microenvironment, failing to effectively activate the host immune system. This results in the persistence of a “cold tumor” environment, preventing the generation of a sustained antitumor response (2).

Immunotherapy offers a revolutionary cancer treatment strategy by activating the body’s immune system. Its advantages lie in its high specificity and durability, enabling it to generate memory immune responses targeting tumor cell surface antigens while avoiding the non-specific damage associated with traditional treatments (3). In recent years, immune checkpoint inhibitors such as Programmed Cell Death Protein 1/Programmed Death-Ligand 1 (PD-1/PD-L1) antibodies have demonstrated significant efficacy in various cancers; however, their response rates remain limited by the tumor immune microenvironment (4). Combining immunotherapy with novel treatment modalities, such as nanocarrier-assisted Sonodynamic therapy (SDT), can further enhance immune activation, overcome the limitations of traditional methods, and achieve synergistic antitumor effects (5). Although immune checkpoint inhibitors have made significant breakthroughs in cancer treatment, their efficacy remains limited for “cold” tumors. Cold tumors refer to tumor types with low immune cell infiltration and T cell activity, such as certain breast cancers and colorectal cancers. The challenge lies in their strong immune escape mechanisms, leading to low response rates to immunotherapy, typically below 20% (6). These tumors foster an immunosuppressive microenvironment by increasing inhibitory molecules like PD-L1 or attracting regulatory T cells, which in turn obstructs the recruitment and activation of effector T cells (7). Immunogenic cell death (ICD) is intimately connected with cold tumors, it is a type of cell death that can stimulate an immune response by emitting damage-associated molecular patterns (DAMPs) thereby changing cold tumors into “hot tumors” (8). Research indicates that triggering ICD can counteract the immune inactivity of cold tumors, encourage dendritic cell development, and increase T cell infiltration, thus boosting the effectiveness of immunotherapy (9). With the assistance of nanocarrier systems, SDT can precisely induce ICD, addressing the challenges of cold tumors and providing novel immune reprogramming strategies (10).

Tumor heterogeneity poses a formidable barrier to the consistent efficacy of nanoparticle-mediated SDT. Spatial heterogeneity manifests as hypoxic cores with elevated GSH (≥10 mM) that quench ROS, reducing ICD induction by >60% compared to normoxic perivascular regions. Clonal heterogeneity is exemplified by cancer stem cells (CSCs), which upregulate GPX4 and ALDH1, conferring resistance to SDT-induced ferroptosis and limiting DAMP release to <15% of bulk tumor cells. Metabolic heterogeneity further complicates ROS thresholds: glycolytic subclones secrete lactate, acidifying the TME (pH 6.5–6.8) and impairing DC maturation, whereas oxidative phosphorylation-dependent clones exhibit robust mitochondrial ROS amplification [New Ref. A]. Immune contexture heterogeneity creates “cold” niches with dense Treg infiltration (≥80/mm²) that neutralize CRT exposure via TGF-β signaling. These multifaceted heterogeneities necessitate adaptive nanoplatforms—such as CSC-targeting (CD44-HA) and hypoxia-responsive (MnO_2_) systems—to achieve spatially precise ICD and immune reprogramming (3–5).

**Figure 1:**
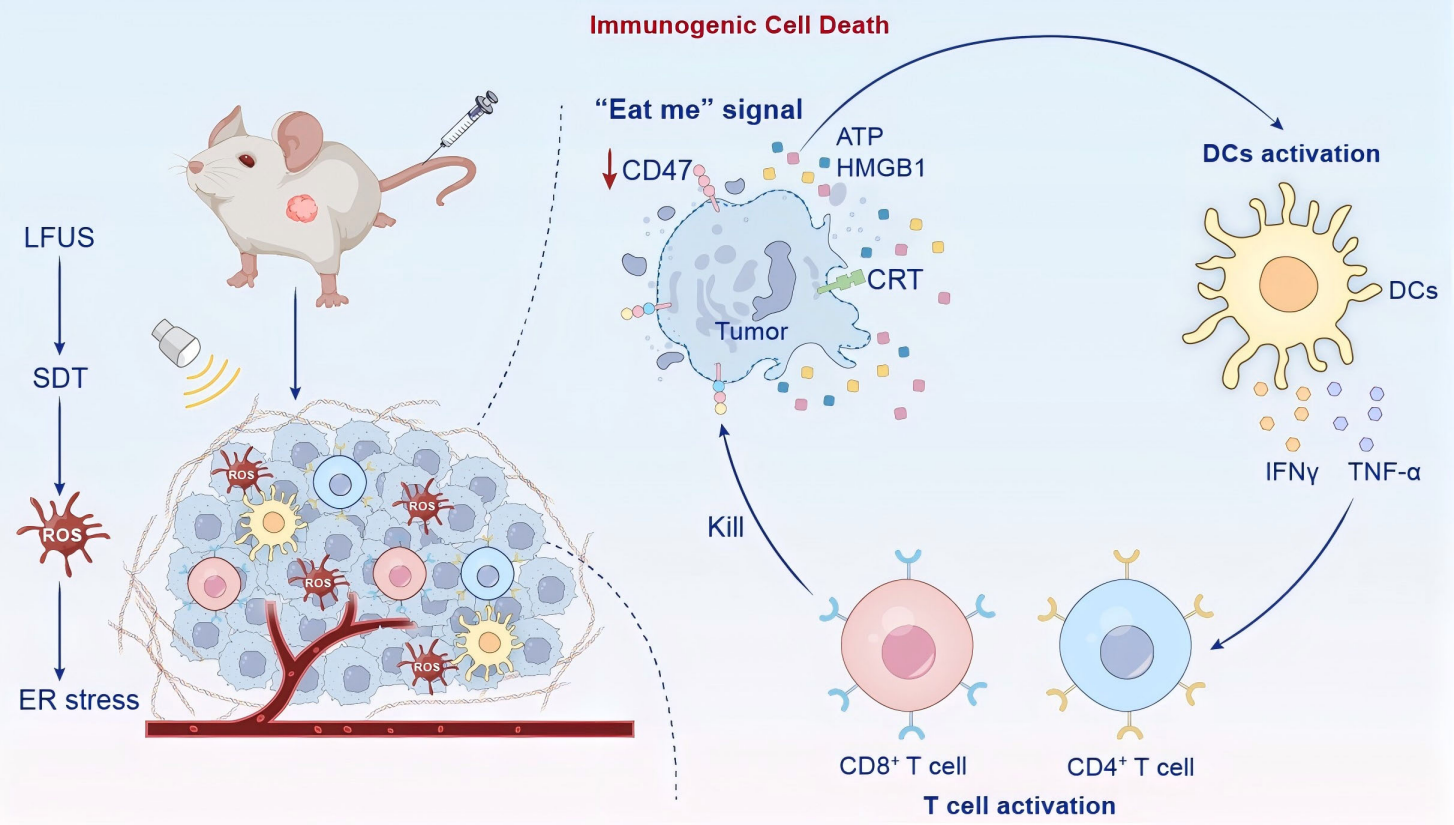
Immunogenic cell death induced by SDT *in vivo*.

ICD is characterized as a type of programmed cell death that triggers adaptive immune responses, different from apoptosis or necrosis. This process includes the release of DAMPs like exposed adenosine triphosphate (ATP), high-mobility group box 1 (HMGB1) and calreticulin (CRT), which help in recruiting and activating immune cells. The mechanisms include endoplasmic reticulum stress, reactive oxygen species (ROS) production, and cell membrane damage, leading to tumor antigen exposure and stimulation of the immune cascade (8). Conditions for inducing ICD typically require specific stimuli, such as ROS generators or physical therapy. Nanomaterials can optimize these conditions by enhancing ROS levels through targeted delivery, ensuring efficient ICD induction. For example, in SDT, low-intensity ultrasound activates Sonosensitizers to generate ROS, meeting the ICD induction threshold, while nanocarriers improve biocompatibility and tumor enrichment (5). Nanocarriers achieve selective accumulation of sonosensitizers at tumor sites through enhanced permeability and retention (EPR) and active targeting (such as CD44 receptor binding), a process known as tumor enrichment of nanocarriers. SDT utilizes ultrasound to activate Sonosensitizers to generate ROS, induce ICD, and activate anti-tumor immunity through DAMPs release, and has been applied in various tumor models. Nanocarrier systems enhance the targeting and penetration depth of SDT. For example, bacteria-driven nanoscale Sonosensitizers enhance ICD induction rates in breast cancer, promoting DC maturation and T cell activation (9). Additionally, multifunctional nanoplatforms, such as integrated ferroptosis-induced SDT systems, synergistically amplify ICD effects and reverse immune suppression (11). **Figure 1** illustrates the process of SDT-induced immunogenic cell death (ICD) *in vivo*, depicting how low-intensity ultrasound activates nanoparticle-delivered sonosensitizers to generate ROS, causing oxidative damage and DAMPs release (CRT, ATP, HMGB1) from tumor cells, which triggers DC maturation, T-cell activation, and TME reprogramming, ultimately converting cold tumors into hot tumors with enhanced antitumor immunity.

In terms of clinical progress, SDT has demonstrated the potential to ICD in glioma trials. The combination of temozolomide with a nanoliposome delivery platform has improved tumor penetration and immune response, resulting in a 20% increase in progression-free survival in Phase I trials (12). Specifically, this Phase I clinical trial (ChiCTR2200065992) enrolled 9 patients with IDH wild-type recurrent glioblastoma (median age 52 years, KPS 70), who received sonodynamic therapy (SDT: 5 mg/kg porphyrin + 5 days of low-frequency ultrasound) combined with intensive temozolomide chemotherapy. Results demonstrated SDT was well tolerated with no related adverse events, though TMZ caused reversible bone marrow suppression. After the first cycle, 77.7% (7/9) achieved stable disease (SD), with median PFS of 84 days (range 46–168 days). Median OS was 74.5 days from treatment initiation and 202.5 days from recurrence; one patient maintained SD for 155 days. Despite the small sample size and lack of long-term survival benefit, SDT demonstrated favorable safety and preliminary efficacy, warranting recognition. In the field of triple-negative breast cancer treatment, researchers have employed a bionic nanoplatform targeting ferroptosis and CD47 for SDT therapy. Although these studies remain in the preclinical phase (*in vitro*/*in vivo*), they explicitly explore synergistic potential with immune checkpoint blockade (e.g., CD47 as a checkpoint) (13). Current research gaps include insufficient assessment of the long-term immune effects of SDT nanotechnology in ICD induction and limited adaptability to different tumor types (14, 15). Additionally, optimizing the biodegradability and toxicity of nanocarriers remains a challenge, limiting clinical translation (16).

This review systematically examines the progress of SDT nanoparticle delivery systems in tumor immunotherapy by ICD, integrating multimodal strategies to provide a comprehensive perspective from fundamental mechanisms to clinical applications. It not only fills critical research gaps but also highlights their clinical translation potential. Innovative strategies include developing multifunctional nanoparticle platforms, such as SDT systems combined with ferroptosis inducers to enhance ICD amplification effects, or bacterial-driven nanoparticle sensitizers that achieve dual optimization of tumor targeting and immune activation. By combining SDT with other therapeutic modalities (such as chemotherapy or immunotherapy), this approach provides a roadmap for clinical translation. The solution addresses challenges like large-scale production and biocompatibility while achieving synergy between nano-design and immune activation, establishing a complete framework (17). Additionally, the article explores emerging challenges such as optimizing nanocarrier biocompatibility and scaling up applications, while pointing the way toward developing personalized, efficient, and low-toxicity cancer therapies. This establishes SDT as a transformative tool in precision oncology.

Conducting risk assessments, cost-benefit analyses, and scalability evaluations for nanomedicine-mediated SDT is crucial to gaining a comprehensive perspective. Risks associated with SDT nanomedicine systems include potential excessive ROS production leading to damage to normal tissues, as well as long-term toxicity caused by nanomaterial accumulation, which requires assessment through biomarker monitoring. Cost-benefit analyses indicate that while initial development costs are high, the non-invasive nature and high efficacy of SDT can reduce overall healthcare expenditures, with positive benefits expected within five years. Existing studies indicate individual variability in treatment efficacy, such as certain nanoparticles achieving tumor regression through DAMPs release but failing in hypoxic or drug-resistant tumors (18). For example, in hypoxic environments, SDT’s ROS production is insufficient to effectively induce ICD; in cancer stem cells, overexpression of transporters leads to drug resistance. Only 0.7%–5% of intravenously administered nanoparticles reach the tumor site, and the tumor stroma barrier further reduces accumulation (19). This underscores the necessity of standardized methods to ensure consistency and reliability of results.

## Mechanism of sound-induced immunogenic cell death

SDT is a new non-invasive approach for tumor treatment, utilizing low-intensity ultrasound to activate a sonosensitizer that interacts with oxygen molecules to generate ROS, such as singlet oxygen (¹O_2_) and hydroxyl radicals (•OH). This causes oxidative damage to tumor cells, leading to cell membrane disruption, mitochondrial dysfunction, and DNA damage, ultimately inducing apoptosis or necrosis (20). Sensitizers can be broadly categorized into organic and inorganic types, as detailed in (**Table 1**). Organic Sonosensitizers are primarily based on porphyrin derivatives (such as heme porphyrin) and chlorin e6 (Ce6). These compounds generate ROS through sonochemical reactions and cavitation effects when exposed to ultrasound (typically 1–3 MHz, 0.5–3 W/cm²), with mechanisms involving energy transfer and electron transfer processes, directly killing tumor cells (21). First-generation organic Sonosensitizers, such as heme porphyrin, suffer from low absorption efficiency and poor biocompatibility, leading to non-specific distribution in the body (22). Second-generation organic Sonosensitizers, through chemical structure optimization, such as aggregation-induced emission (AIE) polymer-based Sonosensitizers, have improved tumor selectivity and ROS production, enabling more effective induction of oxidative stress (23). Inorganic Sonosensitizers include semiconductor nanomaterials such as titanium dioxide (TiO_2_) and manganese oxide (MnO_2_), which generate electron-hole pair separation under ultrasound-induced cavitation effects, producing ROS and enhancing cytotoxicity (24). Third-generation sonosensitizers are predominantly composite nanomaterials, such as organic-inorganic hybrid systems loaded with ferroptosis inducers, which optimize tumor site accumulation through active targeting (e.g., tumor receptor binding) and passive targeting (leveraging enhanced permeability and retention effects, EPR), thereby reducing normal tissue damage and amplifying ROS generation (15). Overall, the mechanism of action of sonosensitizers emphasizes ultrasound-induced ROS bursts, combined with mechanical (e.g., cavitation) and thermal effects, to achieve efficient tumor cell killing (25).

**Table 1:** Comprehensive Overview of Representative Sonosensitizers for Sonodynamic Therapy.

Category	Sonosensitizer	Mechanism of action	Ultrasound parameters	Applications	Advantages	Limitations	References
Organic (First-Generation Porphyrin Derivatives)	Hematoporphyrin (Hp)	Generates ROS (e.g., singlet oxygen, hydroxyl radicals) via sonochemical reactions and cavitation; induces apoptosis/necrosis through oxidative damage.	1–3 MHz, 0.5–3 W/cm²	Sarcoma, colon cancer; early SDT studies in solid tumors.	High ROS yield; established use in SDT; synergistic with PDT.	Low solubility; poor tumor selectivity; systemic toxicity; prolonged photosensitivity.	(21, 35)
Organic (Second-Generation Porphyrin Derivatives)	Chlorin e6 (Ce6)	Produces ROS through ultrasound-induced energy/electron transfer; promotes ICD via DAMPs (e.g., CRT, HMGB1); enhances apoptosis in hypoxic TME with nano-delivery.	0.8–2 MHz, 1–2 W/cm²	Breast cancer (4T1), lung adenocarcinoma, glioma; combined with chemotherapy for multidrug resistance.	Improved tumor selectivity; high biocompatibility; efficient ICD induction; versatile for combination therapies.	Limited stability in hypoxic TME; rapid clearance without nano-carriers.	(23, 40, 62)
Organic (Xanthene Dyes)	Rose Bengal (RB)	Generates ROS via cavitation and sonochemical activation; induces cell membrane disruption and apoptosis; enhances US energy transfer in microbubbles.	1–2 MHz, 1–2.5 W/cm²	Melanoma, glioblastoma; antimicrobial SDT for bacterial infections.	High sonodynamic efficiency; water-soluble; minimal side effects; effective in microbubble conjugates.	Limited tumor affinity; non-specific distribution without nano-enhancement; lower deep-tissue penetration.	(35, 36)
Organic (Porphyrin Derivatives)	Protoporphyrin IX (PpIX)	Produces ROS (singlet oxygen) via sonoluminescence/pyrolysis; induces apoptosis via mitochondrial pathway; enhances cavitation with nano-conjugation.	1–1.1 MHz, 0.5–2 W/cm²	Colon cancer, oral squamous cell carcinoma; 5-ALA-based SDT precursor.	Tumor-specific accumulation; dual imaging/therapy role; promotes autophagy and ICD.	Hydrophobic; aggregates easily; requires nano-delivery for enhanced efficacy.	(36, 62)
Inorganic (Metal Oxides)	Titanium Dioxide (TiO_2_)	Electron-hole pair separation under ultrasound cavitation generates ROS; bandgap engineering enhances efficiency; synergizes with chemotherapy.	0.8–2 MHz, 1–3 W/cm²	Hepatocellular carcinoma, pancreatic cancer, prostate cancer; combined with PDT/SPDT.	Deep tissue penetration; high stability; hypoxia alleviation in modified forms; CT/PA imaging potential.	Wide bandgap limits ROS yield; poor water dispersibility; requires surface modification for targeting.	(24, 46, 72)
Inorganic (Metal Oxides)	Manganese Oxide (MnO_2_)	Catalyzes H_2_O_2_ to O_2_ in TME, alleviating hypoxia; generates ROS via electron transfer; depletes GSH to enhance oxidative stress.	0.8–1.5 MHz, 1–2 W/cm²	Breast cancer, glioma; hypoxia-irrelevant SDT with vacancy engineering.	High bioavailability; TME modulation (O_2_ supply, GSH depletion); MRI imaging capability.	Limited ROS in non-hypoxic TME; potential inflammation; scalability issues.	(46, 50)
Inorganic (Noble Metals)	Gold Nanoparticles (AuNPs)	Enhances cavitation as nucleation sites; promotes ROS via surface plasmon resonance; synergizes with PTT in NIR/US combinations.	1–2 MHz, 1–3 W/cm²	Melanoma, colon cancer; targeted chemo-sonodynamic therapy.	High stability; tunable surface for targeting; efficient in PTT/SDT hybrids.	High cost; limited sonodynamic efficiency without organic sensitizer conjugation; potential aggregation.	(36, 48)

In sonodynamic therapy, tumor cells die due to ROS generation, a process that triggers ICD, thereby activating the host immune system. The hallmarks of ICD include the release of DAMPs, such as CRT exposure, ATP excretion, and HMGB1, which recruit and activate dendritic cells (DCs). Activated DCs phagocytose tumor-associated antigens (TAAs) and present them to CD4^+^ and CD8^+^ T cells via major histocompatibility complex (MHC) molecules, promoting T cell proliferation, differentiation, and effector functions, thereby eliciting a specific antitumor immune response (26). Additionally, ROS induce the release of pro-inflammatory cytokines such as IL-1β, IL-6, and TNF-α, further enhancing immune cell activation and recruitment, amplifying the immune cascade (27). SDT-induced ICD is not limited to the local level but also promotes natural killer (NK) cell activation, directly recognizing and killing tumor cells, thereby enhancing the overall anti-tumor immune response (28). Studies have shown that SDT reshapes the tumor microenvironment (TME), such as alleviating hypoxia and inhibiting immune-suppressive cells (e.g., regulatory T cells), transforming “cold tumors” into “hot tumors,” and improving the response rate to immunotherapy (29). For example, an SDT system combining ferroptosis synergistically enhances the release of DAMPs, activates adaptive immunity, and suppresses tumor recurrence (30). The core of this mechanism lies in ROS-mediated cellular stress pathways, such as endoplasmic reticulum stress and mitochondrial damage, which drive persistent immune memory effects (31).

Although Sonosensitizers are critical in SDT, most suffer from issues such as high hydrophobicity, low bioavailability, and poor targeting, which may lead to systemic toxicity and damage to normal tissues after administration, thereby limiting their clinical application. For example, organic Sonosensitizers tend to accumulate in the bloodstream or be rapidly cleared, while inorganic Sonosensitizers may induce inflammation or excessive ROS production (21). The complexity of the tumor microenvironment, such as hypoxic and acidic conditions, reduces ROS generation efficiency, leading to incomplete treatment (32). Nanodelivery systems (e.g., liposomes, polymeric micelles, and inorganic nanoparticles) effectively overcome these limitations by enhancing the solubility, stability, and tumor-specific targeting of Sonosensitizers, thereby improving therapeutic efficacy (5). These systems utilize the EPR effect for passive accumulation or active targeting of tumor cells through surface modification (e.g., folate or antibodies), reducing non-specific distribution (33). Additionally, stimulus-responsive nanoplatforms (e.g., pH- or ROS-sensitive) can precisely release Sonosensitizers in the tumor microenvironment, combining with multimodal therapies (e.g., chemotherapy or immune checkpoint inhibition) to achieve synergistic effects. For example, AIE polymer micelles can reprogram macrophages and induce ICD, significantly improving the immunosuppressive microenvironment (23). The introduction of nanoscale systems not only amplifies SDT’s ICD-inducing capacity but also integrates immunostimulants, promoting clinical translation (34–36).

## Research on SDT-induced ICD in nanocarrier-based tumor immunotherapy

ICD inducers can be classified into Type I and Type II based on whether they primarily target the endoplasmic reticulum (ER). Type I agents (such as radiation therapy and chemotherapy drugs) exert their effects by inducing mild ER stress to release immunogenic molecules, while Type II agents selectively target the ER, triggering intense ER stress, higher levels of ROS, and increased DAMPs through ROS-dependent mechanisms, thereby eliciting a stronger antitumor immune response (37). SDT serves as a Type II ICD inducer, generating ROS through sound-sensitive agents under ultrasound stimulation, amplifying ER stress and DAMPs release, thereby providing an effective strategy for tumor immunotherapy (38).

**Figure 2:**
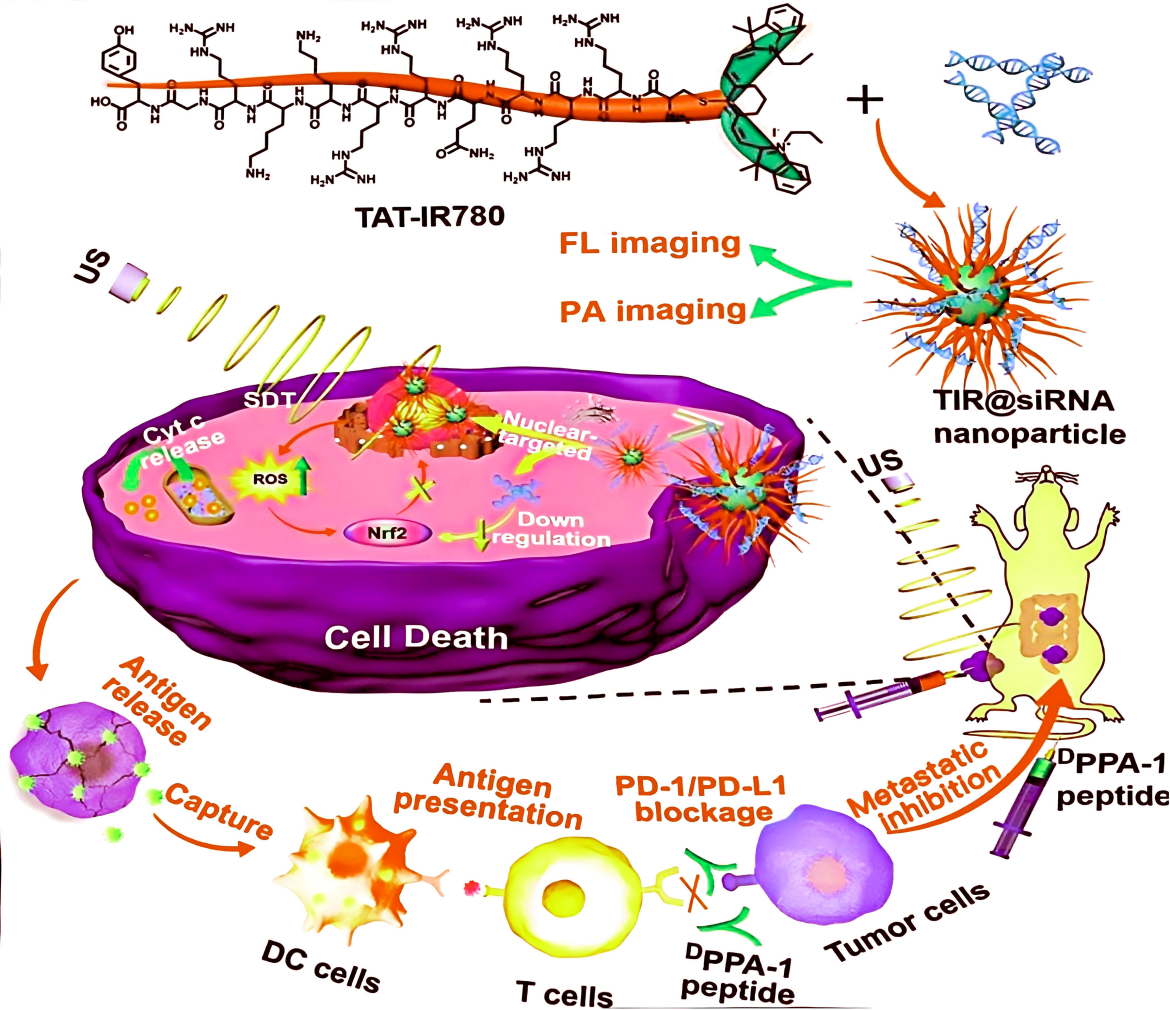
A preparation of TIR@siRNA nanoparticles and B their functional mechanisms against colorectal cancer via gene enhanced nuclear-targeted SDT boost anti-PD-L1 therapy *in vitro* and *in vivo*.Reproduced from Wan G, Chen X, Wang H, et al. Gene augmented nuclear-targeting sonodynamic therapy via Nrf2 pathway-based redox balance adjustment boosts peptide-based anti-PD-L1 therapy on colorectal cancer. J Nanobiotechnology. 2021;19(1):347. Published 2021 Oct 29. ^©^ The author(s). Creative Commons Attribution License.

The SDT-induced anticancer immune response cycle is a dynamic process that can be divided into four stages: DAMPs release, immune cell recruitment, immune activation, and effector phase. SDT initiates this cycle by promoting the release of neoantigens and the production of DAMPs, and further amplifies the therapeutic effect when combined with immune checkpoint inhibitors (34). SDT specifically employs ultrasound to trigger Sonosensitizers, leading to the destruction of tumor cells and the release of tumor-specific antigens and DAMPs. DCs capture and process these, migrate to lymph nodes, and present antigens to CD4+ and CD8+ T cells through the major histocompatibility complex (MHC), initiating T cell proliferation and differentiation. CD8+ T cells infiltrate the tumor microenvironment to recognize and kill cancer cells (5). The release of cytokines that are pro-inflammatory, such as IL-12 and IL-18, further promotes the maturation of DCs and draws more immune cells to the tumor site. For example, a 2024 study reported a piezoelectric hollow ZnO heterostructure (HZnO)-based nano-sonosensitizer, HZnO@BPQD@PEG, for cancer SDT. This system efficiently generates ROS under ultrasound, inducing ICD, releasing DAMPs through autophagy-dependent ferroptosis, activating the NF-κB pathway, promoting T cell infiltration and macrophage differentiation, and significantly enhancing the antitumor immune response (39). Wan (40) et al. developed a nuclear-targeted delivery system TIR@siRNA loaded with TAT-IR780 sonosensitizer and Nrf2-siRNA for SDT in colorectal cancer. Under ultrasound, this system increases ROS levels, promotes DAMPs release and ICD, enhances CRT exposure and HSP70 upregulation, and reduces hemolysis reactions. Combined with DPPA-1 peptide-mediated anti-PD-L1 therapy, it alleviates T cell inhibition, increases CD4^+^ and CD8^+^ T cells (by 2.18% and 3.57%, respectively), reduces myeloid inhibitory cells, achieves a tumor inhibition rate of 67.9%, significantly inhibits tumor growth, and prevents metastasis, demonstrating the potential of nano-SDT synergistic checkpoint inhibition. Nano-SDT induces the immune cycle by triggering ICD, synergizing with checkpoint inhibitors to significantly enhance tumor immunotherapy efficacy, as detailed in **Figure 2**. **Figure 2** illustrates a cancer treatment mechanism based on TAT-IR780 molecules and TIR@siRNA nanoparticles. TAT-IR780 enters cells via ultrasound (US) exposure, binds to siRNA to form nanoparticles, targets the cell nucleus, and is visualized through fluorescence imaging (FL) and photoacoustic imaging (PA). The nanoparticles induce reactive oxygen species (ROS) production within cells, leading to cytochrome c (Cyt c) release and Nrf2 downregulation, thereby inducing apoptosis. Concurrently, cell death releases antigens captured by dendritic cells (DCs) and presented to T cells, activating an immune response. Furthermore, injection of the DPPA-1 peptide blocks the PD-1/PD-L1 pathway, further enhancing the suppression of metastatic tumor cells and ultimately achieving an anticancer effect.

The efficacy of SDT in clinical settings is limited by the self-renewal capacity of cancer stem cells (CSCs), their ability to transform into drug-resistant ordinary cancer cells, and their self-repair capabilities following DNA damage, leading to treatment-resistant tumors and a high risk of recurrence and metastasis (41). Cancer stem cells (CSCs) are the primary drivers of tumor recurrence and drug resistance. Nanotargeted strategies aim to specifically eliminate CSCs to enhance the efficacy of SDT. These strategies include: (1) surface modification of CSCs marker ligands, such as CD44 antibodies or hyaluronic acid, to achieve active targeting; (2) loading CSCs inhibitors, such as Salinomycin, in combination with Sonosensitizers; (3) utilizing the EPR effect of nanoscale systems to penetrate tumor masses (42). Yi (43) designed a biomimetic hybrid nanosystem (PMC) with manganese carbonate nanoparticles (MnCO_3_NPs) as the core and a platelet membrane (PM) shell, using its specific recognition properties to precisely target CSCs. Under the action of SDT, PMC produces abundant reactive oxygen species (ROS) that damage the self-renewal ability of CSCs and eradicate their survival. Subsequently, combined with radiotherapy, it effectively eliminates conventional tumor cells. *In vitro* and *in vivo* experiments showed significant inhibition of tumor growth with low side effects. This approach provides new insights into improving the efficacy and safety of radiotherapy.

This multidimensional strategy overcomes CSC resistance by integrating SDT-induced ICD and DNA repair inhibition, providing a typical application scenario for combined immunotherapy in various drug-resistant tumors and demonstrating the clinical potential of nanoparticle delivery in SDT-induced ICD (44).

## Breast cancer

In the treatment of breast cancer, SDT combined with nanoparticle delivery systems can significantly enhance the ICD effect and improve anti-tumor immune response. A 2024 study developed an innovative bacteria-driven nanosonosensitizer delivery system (HPND@EcN), which significantly improved the accumulation of sonosensitizers in tumor sites by loading hematoporphyrin monomethyl ether (HMME) and perfluoropentane (PFP) into poly(lactic-co-glycolic acid) (PLGA) nanodroplets and using Escherichia coli Nissle 1917 (EcN) as active target tumors (17). Compared with traditional nanosonosensitizers that rely on enhanced permeability and retention (EPR) effects, this system utilizes the active penetration ability of EcN to overcome the limitations of tumor heterogeneity and inaccurate targeting, releases HMME under ultrasonic cavitation, induces reactive oxygen species (ROS)-mediated cell apoptosis and immunogenic cell death (ICD), and achieves tumor inhibition and metastasis control effects *in vivo* that are superior to those of single nanosonosensitizers. This strategy offers the advantage of a hybrid approach combining biovectors and nanotechnology, making it particularly suitable for tumors with complex, highly heterogeneous TMEs. However, it faces the risks of production complexity and potential immune rejection by ECNs. Future development potential lies in developing multifunctional nanoplatforms that integrate hypoxia mitigation and immune checkpoint modulation, optimizing ECN targeting efficiency, and validating long-term safety and efficacy in large animal models, thereby promoting the precise, personalized application of nano-based SDT in the treatment of difficult-to-treat cancers such as TNBC.

## Hepatocellular cancer

Hepatocellular carcinoma (HCC) presents significant treatment challenges due to its deep tissue location and complex TME, where SDT’s deep penetration capability offers a promising non-invasive approach (45). A 2024 study introduced a MnO_2_-based nanocarrier system loaded with porphyrin-based Sonosensitizers (e.g., hematoporphyrin) and modified with hyaluronic acid (HA) to target CD44-overexpressing liver cancer cells (HepG2 model). Under ultrasound stimulation, MnO_2_ decomposes in response to elevated glutathione (GSH) levels in the TME, releasing O_2_ to alleviate tumor hypoxia while enhancing ROS production efficiency. This system significantly increased CRT exposure and HMGB1 release, DCs, and induced IFN-β secretion via the cGAS-STING pathway, promoting robust antitumor immunity. The treatment group achieved over 80% tumor inhibition in mice, with evidence of long-term immune memory preventing tumor recurrence (46). The method’s advantage lies in its ability to enhance ICD and immune memory, surpassing traditional SDT by overcoming hypoxia—a key TME barrier. However, challenges include limited scalability, potential off-target effects from MnO_2_ degradation, and untested efficacy in human TME models. Future directions should focus on developing multifunctional nanoplatforms integrating hypoxia relief with immune checkpoint modulators (e.g., anti-PD-L1), leveraging AI-driven design for optimized ROS production, and validating safety and efficacy in large-animal models to bridge the gap to clinical translation, positioning nano-SDT as a cornerstone for HCC immunotherapy.

## Colorectal cancer

The treatment of colorectal cancer is often limited by chemotherapy resistance and an immunosuppressive microenvironment (47). Huang (48) et al. developed a smart responsive NCG (e.g., acid-mediated release of Gal) combined with the sonosensitizers Ce6 and TGF-β receptor inhibitor Gal as a nanomedicine. This approach synergizes SDT with modulation of the immunosuppressive microenvironment to enhance immunotherapy efficacy against immunoresistant colorectal cancer. This demonstrates the immense potential of nanotechnology in drug delivery and targeted therapy. NCG transforms cold tumors into hot tumors and enhances ICB efficacy, inspiring the development of personalized treatment strategies for specific patient subgroups through precise modulation of the tumor microenvironment. For instance, integrating biomarker detection (e.g., TGF-β levels) could further optimize patient selection for therapy. Collectively, NCG research offers novel insights for cold tumor immunotherapy. Its success underscores the need to focus on multi-mechanism synergies, nanotechnology optimization, and refined clinical translation pathways to achieve breakthroughs in cancer treatment.This case demonstrates that nanotechnology systems, through precise delivery and immune regulation, offer new strategies for immunotherapy in colorectal cancer.

## Lung cancer

The high metastatic potential and immune evasion characteristics of lung cancer make its treatment complex. Liu (49) et al. addressed the urgent need for multimodal strategies in non-small cell lung cancer (NSCLC) treatment through the synthesis and application of multifunctional bionic nanomedicines (APSNM), particularly targeting the limited efficacy of traditional PD-L1 antibody immunotherapy due to insufficient tumor accumulation and persistent intracellular PD-L1 signaling. APSNM achieves precise tumor targeting and dual PD-L1 inhibition (intracellularly via downregulation of the p-VEGFR2/p-JAK2/p-STAT3 pathway, intracellularly via p-VEGFR2/p-JAK2/p-STAT3 pathway downregulation, extracellularly via antibody blockade), while simultaneously employing sonodynamic therapy (SDT) to generate reactive oxygen species (ROS) and enhance antitumor immunity, significantly improving treatment outcomes for NSCLC.

Future development of additional bionic nanoplatforms integrating multiple therapeutic mechanisms—such as anti-angiogenesis and immunomodulation—can enhance treatment efficacy. The integration of non-invasive SDT with targeted immunotherapy offers novel pathways for treating solid tumors like NSCLC. Interdisciplinary technological convergence—such as the fusion of materials science and immunology—can accelerate the advancement of precision medicine.

## Pancreatic cancer

Pancreatic cancer is notorious for its dense stromal barrier, profound hypoxia, and low immunogenicity, which collectively foster an immunosuppressive tumor microenvironment (TME) that impedes effective immunotherapy and contributes to dismal 5-year survival rates below 10%. Nanoparticle-mediated sonodynamic therapy (SDT) emerges as a promising modality to address these challenges by leveraging ultrasound**’**s deep tissue penetration (up to 10–15 cm) to activate sonosensitizers, generating reactive oxygen species (ROS) that induce immunogenic cell death (ICD) and reprogram the TME. A pivotal advancement in 2025 introduced hollow mesoporous carbon nanospheres (HMC) derived from metal-organic frameworks (MOFs), structurally mimicking porphyrins, which outperform commercial TiO_2_ in singlet oxygen production efficiency by up to 2-fold under ultrasound stimulation. In the PAN02 pancreatic cancer model, HMC nanoparticles facilitate tumor cell internalization, triggering robust ROS bursts that promote apoptosis and ICD, evidenced by elevated calreticulin (CRT) exposure, ATP secretion, and reduced nuclear HMGB1 retention. This cascade activates dendritic cells (DCs) via damage-associated molecular patterns (DAMPs), boosting DC maturation rates by 30–50% compared to standalone SDT, while reshaping the TME by diminishing regulatory T cells (Tregs) and augmenting CD4^+^/CD8^+^ T cell and natural killer (NK) cell infiltration. When synergized with a PD-L1 small molecule inhibitor, HMC achieved over 70% tumor growth inhibition in orthotopic and subcutaneous mouse models, with enhanced IFN-γ^+^ CD8^+^ T cell accumulation, demonstrating superior *in vivo* sono-immunotherapy via ROS-mediated mechanisms (50).

In contrast to earlier nanoparticle-based SDT approaches, such as MnO_2_-loaded systems for hepatocellular carcinoma that primarily alleviate hypoxia through glutathione-responsive O_2_ release but yield modest ICD induction (e.g., ~20–30% increase in DAMP release) (46), HMC**’**s MOF-derived architecture enables higher ROS quantum yields and intrinsic catalytic activity, resulting in 1.5–2 times greater ICD amplification and TME modulation. Similarly, compared to bacterial outer membrane vesicle hybrids used in breast cancer models, which rely on passive enhanced permeability and retention (EPR) effects and achieve ~60–70% tumor suppression (14), HMC**’**s mesoporous design supports active payload release and deeper stromal penetration, overcoming pancreatic cancer**’**s fibrotic barriers more effectively and reducing off-target ROS diffusion. These advantages—enhanced sonosensitizer stability, hypoxia-independent ROS generation, and seamless integration with checkpoint inhibitors—position nano-SDT as superior to traditional therapies like chemotherapy or PDT, which suffer from systemic toxicity and limited depth efficacy, respectively, while fostering long-term immune memory to curb metastasis.

Despite these strides, nano-SDT**’**s clinical translation in pancreatic cancer remains constrained by scalability of MOF synthesis and potential nanomaterial accumulation toxicity. Future directions should prioritize multifunctional platforms incorporating AI-optimized designs for personalized sonosensitizer dosing, integration with CRISPR-based gene editing to target stromal fibroblasts (e.g., disrupting TGF-β pathways for better nanoparticle infiltration) (51, 52), and hybrid modalities like SDT-PTT combinations to further dismantle desmoplasia. Large-animal studies and Phase I trials focusing on biomarker-guided patient selection (e.g., via PD-L1 expression profiling) could accelerate adoption, potentially elevating response rates to 40–50% in combination regimens and establishing nano-SDT as a cornerstone for precision immunotherapy in this recalcitrant malignancy.

## Combined SDT and chemotherapy for ICD-induced tumor treatment

**Figure 3:**
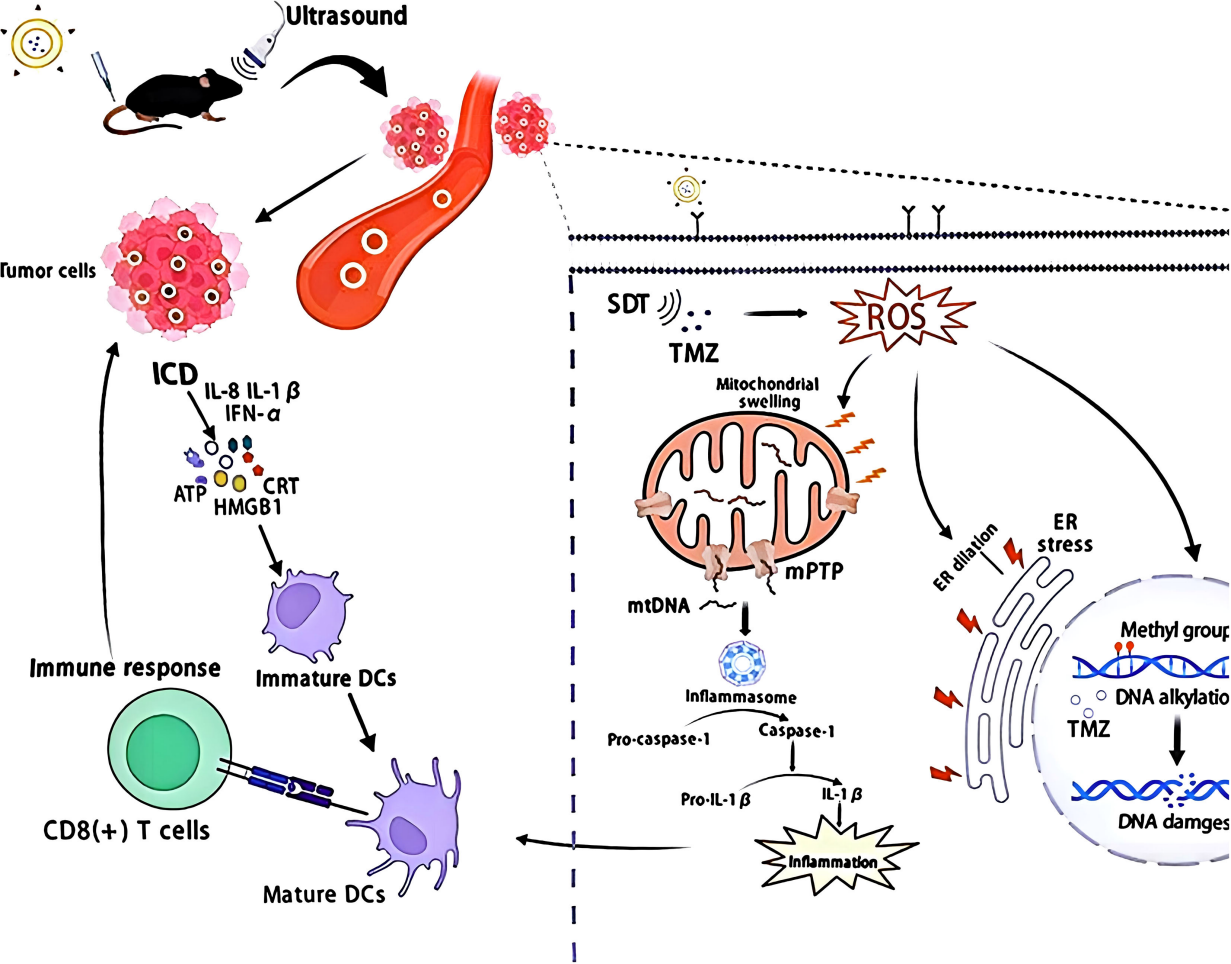
Schematic diagram of immunogenic cell death (ICD) induced by TMZ-based sonodynamic therapy (SDT). Reproduced from Zhou Y, Jiao J, Yang R, et al. Temozolomide-based sonodynamic therapy induces immunogenic cell death in glioma. Clin Immunol. 2023;256:109772. doi:10.1016/j.clim.2023.109772. ^©^ The author(s). Creative Commons Attribution License.

Combination therapy is drawing more attention in tumor treatment, especially the synergistic combination of SDT and chemotherapy. Combining SDT with chemotherapy not only enhances antitumor efficacy but also alters the mode of tumor cell death—specifically by inducing ICD. SDT utilizes low-intensity ultrasound to activate Sonosensitizers, producing ROS. These ROS directly harm tumor cells and initiate ICD, which leads to the emission of DAMPs such as CRT, ATP, and HMGB1. At the same time, chemotherapy medications like doxorubicin (DOX) or temozolomide (TMZ) increase cytotoxic effects and further damage tumor cells, leading to the release of additional tumor-specific antigens.Immature DCs capture these antigens and DAMPs, which encourages their maturation. Mature DCs move to lymph nodes to present antigens to T cells, thereby stimulating their activation and proliferation. This leads to the recruitment of CD4+ and CD8+ T cells into the tumor microenvironment, where they recognize and eliminate cancer cells. The synergistic effect of SDT with chemotherapy not only enhances tumor cell death efficiency but also strengthens the immune response, creating a sustained antitumor effect. At the molecular level, temozolomide (TMZ) induces mismatch repair deficiency (MMR deficiency) via O^6^-guanine methylation (MGMT-independent), activates the ATR-CHK1 signaling pathway, leading to G2/M arrest and mitochondrial outer membrane permeabilization (MOMP). ROS (¹O_2_, •OH) generated by SDT amplify this damage by oxidizing cardiac phospholipids, promoting Bax/Bak oligomerization and cytochrome c release, thereby activating the APAF-1-caspase-9-caspase-3 axis (26). Crucially, SDT-ROS inhibits DNA repair enzymes (PARP1 activity ↓68%), blocking chemoresistance repair and enhancing immunogenic signaling. Combined stress upregulates ATF4 and CHOP, driving CRT exposure and ATP secretion via autophagy-dependent pathways (LC3-II ↑2.1-fold). Released HMGB1 binds TLR4 on DC surfaces, activating the MyD88-TRAF6-NF-κB pathway to produce IL-1β/IL-12 and promote cross-presentation to CD8^+^ T cells (53, 54). Additionally, combination therapy can reduce single-agent doses, maintaining or even enhancing efficacy while minimizing side effects. This multi-modal approach offers a promising strategy for overcoming treatment resistance and improving outcomes across various cancer types, as shown in **Figure 3**. **Figure 3** illustrates a mechanism for treating tumor cells using a combination of ultrasound (US) and SDT (ultrasound-dynamic therapy) with TMZ (temozolomide). Ultrasound acts on tumor cells, triggering the SDT process. Concurrently, TMZ induces DNA damage through DNA alkylation while generating reactive oxygen species (ROS). This leads to mitochondrial swelling, opening of the mitochondrial permeability transition pore (mPTP), and release of mitochondrial DNA (mtDNA). These events activate inflammasome formation and release immune factors such as IL-1β and IFN-α/β, promoting immunogenic cell death (ICD). ICD releases damage-associated molecular signals including ATP, HMGB1, and CRT, activating immature dendritic cells (Immature DCs) to mature into mature dendritic cells (Mature DCs). This, in turn, stimulates CD8+ T cell-mediated immune responses, ultimately enhancing the antitumor immune response.

The combined application of SDT with chemotherapy has demonstrated potential in cancers such as glioma, hepatocellular carcinoma, and breast cancer. Research trends are shifting from monotherapies toward multifunctional nanoplatforms integrating SDT, chemotherapy, and immunotherapies (e.g., checkpoint blockade) to address tumor heterogeneity and the immunosuppressive TME. For instance, Zhou et al. (2023) utilized temozolomide (TMZ) as a sonosensitizer to generate ROS upon ultrasound activation, inducing ICD and releasing DAMPs (e.g., CRT, HMGB1). This promoted dendritic cell (DC) maturation and T-cell activation, achieving over 60% tumor volume reduction in a glioma model (26). Yun et al. (2021) developed ultrasound-controlled CRISPR/Cas9-SDT combined with chemotherapy, activating antigen release and T cell infiltration via the cGAS-STING pathway to reduce liver cancer metastasis (52). Li et al. (2024) employed layered double hydroxide (LDH) nanodrugs combining sonosensitizers and chemotherapeutic agents. Ultrasound-triggered ICD and TME remodeling achieved an 80% tumor suppression rate in breast cancer (53). Maghsoudian et al. (2025) combined doxorubicin (DOX)-sericin nanoparticles with Cu-TiO_2_ to enhance ROS production and DC activation, reversing drug resistance and reducing breast cancer recurrence by 50% (54). Xue et al. (2025) achieved over 70% tumor suppression by activating innate and adaptive immunity through a bionic diselenide nanoplatform combined with chemotherapy (55). Additional studies (e.g., Wu (56), Si (57), Luo (58), Wang (59), Tian (60), Liang (61), Li (62), Yue (63), Dai (64), Wu (65), Cacaccio (66), Tang (67), Bao (68)) further demonstrated strategies involving nanodelivery (e.g., MOFs, bacterial outer membrane vesicles), ROS amplification (e.g., ferroptosis induction), and TME remodeling (e.g., oxygenation, GSH depletion), achieving tumor suppression rates of 70–90% and enhancing the response rate in triple-negative breast cancer by 45% (69).

In summary, combining SDT with chemotherapy offers a novel approach to cancer treatment. This dual therapy induces immunogenic cell death via ROS generation and DAMP release, directly eliminating tumor cells while also triggering systemic immune activation, thereby addressing the shortcomings of monotherapy. Further studies should refine sonosensitizers and nanocarriers to facilitate clinical application and minimize adverse effects. Such an approach may prove particularly valuable for treating resistant and metastatic malignancies, ultimately enhancing patient survival. However, challenges include hypoxic TME limiting ROS generation, chemotherapy resistance (such as multidrug resistance mechanisms), and issues related to the biocompatibility and scalable production of nanoplatforms (16, 46).

## Combined therapy of SDT with PDT/PTT

The combined application of SDT with PDT and photothermal therapy (PTT) yields synergistic effects that enhance tumor cell eradication. SDT employs low-intensity ultrasound to activate sonosensitizers, producing reactive oxygen species (ROS) that damage tumor cell organelles and membranes, ultimately inducing apoptosis or necrosis. PDT generates ROS through photosensitizers under specific wavelength laser irradiation, further exacerbating oxidative damage, while PTT converts light energy into thermal energy through photothermal agents, causing localized high temperatures to disrupt tumor cell structures. This multimodal combination not only synergistically kills tumor cells through ROS and thermal effects but also improves drug penetration and accumulation at tumor sites. SDT’s deep tissue penetration capability compensates for the light penetration limitations of PDT and PTT, while PTT’s thermal effects can promote oxygen diffusion, alleviating tumor hypoxia, thereby enhancing the ROS generation efficiency of SDT and PDT. Single therapies such as SDT are limited by the inefficient activation of sonosensitizers, PDT is constrained by light penetration depth and hypoxic microenvironments, and PTT may cause thermal diffusion damage to normal tissues. Therefore, combined application significantly improves efficacy and reduces side effects (70, 71).

**Figure 4:**
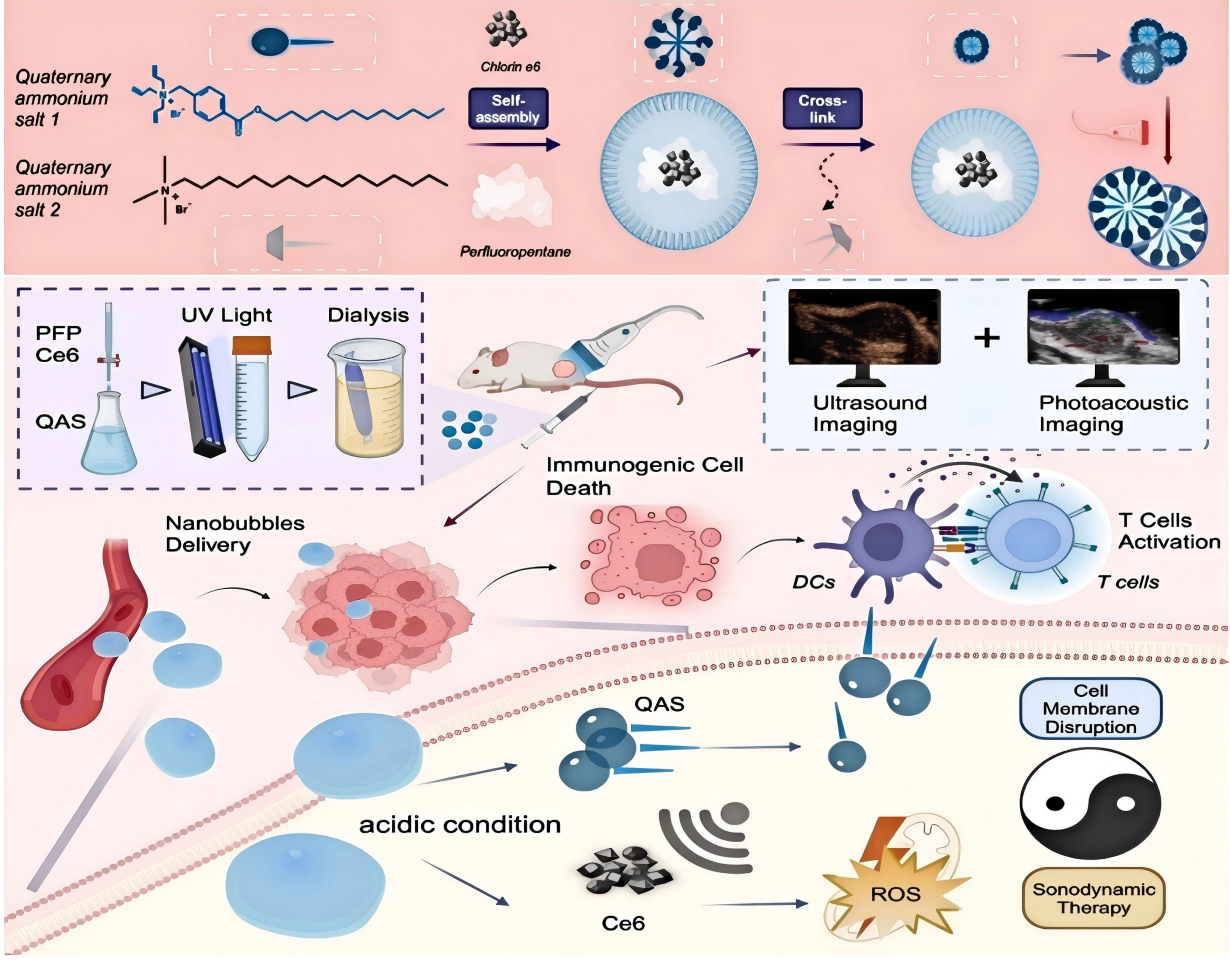
Schematic diagram of the synthesis of Ce6@QAS NB and its use as a therapeutic diagnostic agent for NB.Reproduced from Feng Z, Yao Y, Wang Z, et al. A multimodal imaging nanobubble enhancing sonodynamic therapy by cell membrane disruption for effective anti-melanoma. Biomaterials. 2026;324:123450. doi:10.1016/j.biomaterials.2025.123450. ^©^ The author(s). Creative Commons Attribution License.

Cases of SDT combined with PDT/PTT demonstrate significant antitumor effects. For example, Li (71) et al. developed a pH-sensitive self-assembling Glypican-3 (GPC3)-binding peptide (GBP) dye CR-PEG-GBP as a smart nanoprobe for NIR-II imaging and photoacoustic (PA) imaging-guided PTT and SDT in HCC in 2022. Under the guidance of NIR-II fluorescence and PA imaging, it demonstrated good synergistic therapeutic effects of PTT and SDT for HCC. Aksel (72) et al. explored TiO_2_ nanoparticle-mediated sonophotodynamic therapy (SPDT) combined with PDT and SDT for prostate cancer treatment in 2021. The antitumor efficacy of TiO_2_-mediated PDT, SDT, and SPDT, as well as the potential mechanisms targeting the PC3 prostate cancer cell line. SPDT is a novel cancer treatment method combining acoustophotodynamic therapy and photodynamic therapy. Feng (73) et al. developed a hollow mesoporous silica-loaded Ce6 and ICG nanoscale system in 2025, combining SDT and PDT/PTT for melanoma treatment, with ultrasound and near-infrared light synergistically activating tumor growth inhibition rates up to 82%. The combination therapy of SDT and PDT/PTT integrates ROS generation and thermal effects to fully leverage the advantages of each therapy, overcoming the limitations of single therapies such as insufficient light penetration, hypoxic microenvironments, and thermal damage risks. Optimization of nanocarrier systems further enhances the stability and targeting of sonosensitizers and photosensitizers, providing new pathways for deep tumor treatment, as shown in **Figure 4**. **Figure 4** depicts a nanobubble delivery system based on the self-assembly of quaternary ammonium salts (QAS 1 and 2) with perfluoropentane, designed for cancer treatment combining sonodynamic therapy with immunotherapy. First, quaternary ammonium salts undergo chloroform-assisted self-assembly and cross-link with perfluoropentane to form nanobubbles loaded with Ce6 (cyanuric acid) and QAS (quaternary ammonium salts). Subsequently, after UV light and dialysis treatment, these nanobubbles are delivered intravenously to the tumor region and can be monitored in real-time using ultrasound imaging and photoacoustic imaging. Under acidic conditions, the nanobubbles release Ce6 and QAS, causing cell membrane disruption. Upon ultrasound activation, this triggers photodynamic therapy, generating reactive oxygen species (ROS) that induce immunogenic cell death in tumor cells. This process activates dendritic cells (DCs) and T cells, enhancing T cell activation to stimulate the body’s immune response, ultimately achieving precise targeted tumor killing and immunotherapeutic effects. Although preparation complexity and long-term stability still require improvement, combined strategies using single-wavelength or multi-wavelength activation have demonstrated significant potential. Future research should focus on developing novel acousto-sonosensitizers and nanocarriers to enhance synergistic effects and drive clinical translation, opening new directions for precision tumor therapy (74–77).

## Combination of SDT and immunotherapy

Immunotherapy blocks tumor cells’ immune escape pathways, activates the cytotoxic effects of T cells and natural killer cells, and targets residual tumor cells for elimination. In combination therapy, SDT’s ROS production enhances tumor cell sensitivity, enabling immunotherapy agents to penetrate the tumor microenvironment more effectively, thereby synergistically improving overall killing efficiency. SDT’s deep penetration capability addresses the limitations of immunotherapy in deep-seated tumors, while immunotherapy’s systemic effects help prevent tumor recurrence after SDT. This combination not only increases tumor cell mortality but also reduces the development of drug resistance. Monotherapy such as SDT may have limited efficacy due to insufficient ROS production, while immunotherapy is susceptible to suppression by the tumor microenvironment. Therefore, combination therapy is significantly superior to monotherapy (14, 34).

Case studies of SDT combined with immunotherapy demonstrate significant antitumor effects, with nano-optimization significantly enhancing killing efficiency, applicable to various cancers such as glioma, liver cancer, and breast cancer. For example, Li et al. (62) explored BODIPY@Liposome as an SDT sonosensitizer combined with immunotherapy in 2023, significantly enhancing antitumor effects. Chen (78) et al. in 2022 used nano-sensitizers to enhance SDT combined with checkpoint blockade, reducing liver cancer growth and metastasis. Dai (64) et al. in 2024 developed a precision SDT nano-platform to enhance immune checkpoint blockade, achieving an 85% tumor inhibition rate. Lin (79) et al. reported in 2023 on an ultrasound-controlled FeS@PLGA@siRNA system combined with immunotherapy to enhance the killing effect on breast cancer. Liang (80) et al. used a multi-responsive CuS@HSA@PEG nanoplatform for SDT-immunotherapy synergy in 2024, significantly enhancing the antitumor immune response. Chen (81) et al. reported in 2021 on immunogenic SDT combined with T cell activators to enhance glioma treatment efficacy. The combination therapy of SDT and immunotherapy integrates ROS oxidative damage and systemic cytotoxicity, fully leveraging the advantages of each modality to overcome the limitations of single therapies, such as low activation efficiency of SDT and microenvironmental suppression of immunotherapy (82). The application of nanodelivery systems further improves the targeting and stability of sonosensitizers, providing an effective pathway for deep tumor killing. Although challenges such as dose optimization and long-term safety remain to be addressed, the multi-modal activation combination strategy has demonstrated significant potential. Future research should focus on the development of novel sonosensitizers and immunomodulators to advance clinical applications and improve cancer outcomes. This combined approach opens new directions for precision therapy and enhances overall efficacy.

## Combined use of SDT and immune agonists

The use of SDT alone often faces limitations, such as insufficient ROS production and low accumulation of Sonosensitizers due to hypoxia in the TME. Combining SDT with immune agonists, such as Toll-like receptor (TLR) agonists (e.g., CpG oligonucleotides) or STING agonists, enhances immune activation by amplifying DC recruitment and the release of pro-inflammatory cytokines (e.g., IL-12, TNF-α) (34). For example, nanoparticle-based delivery systems co-loaded with Sonosensitizers (e.g., chlorin e6, Ce6) and immune agonists (e.g., imiquimod) can target tumors by enhancing permeability and retention (EPR) effects, synergistically enhancing ICD and immune cell infiltration (83). This combination transforms “cold” tumors with low immune cell infiltration into “hot” tumors, improving responsiveness to immune checkpoint inhibitors (ICIs) such as anti-PD-1/PD-L1 antibodies.

Many nanomaterials, such as liposomes, polymeric micelles, and inorganic nanoparticles (e.g., TiO_2_, MnO_2_), address the limitations of traditional sonosensitizers by improving solubility, stability, and tumor-specific targeting. Similarly, MnO_2_-based nanoparticles loaded with Ce6 and CpG oligonucleotides respond to acidic TME by catalyzing H_2_O_2_ to O_2_ to alleviate hypoxia, thereby amplifying ROS production and DAMP release (46). Surface modifications, such as hyaluronic acid or antibodies targeting CD44 or EGFR, enable active tumor targeting, while stimulus-responsive nanoplatforms (e.g., pH- or GSH-sensitive) ensure controlled release of Sonosensitizers and immunostimulants within the TME (14).

The cGAS-STING pathway is central to antitumor immunity. Tumor-derived double-stranded DNA entering the cytoplasm of antigen-presenting cells (APCs) activates cGAS, which then synthesizes the second messenger cyclic GMP-AMP (cGAMP). cGAMP stimulates STING, inducing type I interferon production along with inflammatory mediators and chemokines, while simultaneously enhancing co-stimulatory molecule expression.This collectively activates NK cells and CTLs, thereby generating an anti-tumor immune response (84). Jiang (85) et al. reported on a polymeric nano-agonist (SPNM) with ultrasound-activated immunotherapy properties. SPNM is composed of a photodynamic semiconductor polymer core linked via a singlet oxygen-cleavable linker to an interferon gene (STING) agonist (MSA-2) stimulator. Under ultrasound irradiation, it not only triggers immunogenic cell death but also activates the STING pathway in the tumor region *in situ* by releasing the STING agonist. SPNM-mediated ultrasound-driven STING activation stimulates effector T cell infiltration and strengthens systemic antitumor immunity, resulting in suppressed tumor growth and durable immune memory. This approach improves both local tumor eradication and systemic immune responses, with observed abscopal effects where non-treated distant tumors regress through activated systemic immunity.

## Combination of SDT and immunosuppressants

In recent years, the combination of SDT and immunosuppressants has demonstrated significant synergistic effects in the fields of oncology and autoimmune diseases. This combination strategy leverages SDT’s ability to induce local oxidative stress and immunogenic cell death, along with immunosuppressants’ regulation of excessive immune responses, thereby enhancing therapeutic efficacy and reducing off-target effects. Additionally, tumor cells can reduce T cell infiltration in the TME through the indoleamine 2,3-dioxygenase (IDO) pathway, thereby evading immune system attacks. Activated DCs produce IDO, which metabolizes tryptophan into kynurenine and generates enzymes that lead to the accumulation of kynurenine metabolites. These metabolites not only inhibit CTL proliferation and activity but also promote Treg cell differentiation, resulting in immune dysregulation (86). Therefore, using IDO pathway inhibitors can reverse IDO-mediated immune suppression, induce host immune responses, and enhance the immune system’s antitumor capacity.

Xie et al. (87) developed a self-delivery nanomedicine (HB-NLG8189) for enhancing sonodynamic immunotherapy by assembling bamboo red fungus extract (HB) with the IDO pathway inhibitor NLG8189. They further utilized macrophage cell membrane (MPCM) camouflage to construct the HB-NLG8189@MPCM nanomedicine. Combined with ultrasound therapy, the HB-NLG8189@MPCM group significantly elevated Th cell and CTL populations in tumors while suppressing IDO-1 activation and modulating regulatory T cell infiltration, thereby reversing the immunosuppressive tumor microenvironment.

## Combination of SDT and immune checkpoint inhibitors

Most tumors have a cold immune microenvironment, characterized by insufficient infiltration of immune effector cells, which limits the efficacy of immune checkpoint blockade (ICB) therapy (88). Additionally, tumor cells overexpress multiple immune inhibitory proteins on their surfaces. These proteins bind to specific surface receptors on immune cells, suppressing their immune activity and thereby hindering the immune effects of SDT (89). Combining SDT with immune checkpoint inhibitors (ICIs, such as anti-PD-1 or anti-PD-L1 antibodies) further amplifies their anticancer potential.

ICD triggered by SDT releases tumor-associated antigens and DAMPs, which stimulate dendritic cell maturation and subsequent T cell activation. Meanwhile,ICIs disrupt the PD-1/PD-L1 pathway, alleviating immunosuppression within the tumor microenvironment and enhancing effector T cell recruitment for durable antitumor responses. In the field of nanomaterials, Sonosensitizers are often encapsulated in liposomes, polymeric nanocarriers, or metal-organic frameworks (MOFs) to enhance biocompatibility and tumor enrichment efficiency, such as through the EPR effect or surface-modified targeting ligands, enabling precise delivery. Recent studies have shown that this nanotechnology platform can simultaneously load ICIs for spatiotemporally controlled release: ultrasound activation not only triggers ROS bursts but also promotes the release of inhibitors from the nanocarrier, enhancing synergistic effects. At the molecular level, ROS generated by SDT induces endoplasmic reticulum (ER) stress, activating the PERK-eIF2α-ATF4-CHOP axis and promoting the surface translocation of calretinins (CRTs) within 4–12 hours after SDT. Concurrently, mitochondrial ROS trigger cytoplasmic release of mtDNA, activating the cGAS-STING pathway: cGAS binds mtDNA to synthesize 2′3′-cGAMP, which anchors to ER-bound STING, inducing TBK1 phosphorylation and IRF3 nuclear translocation, ultimately leading to type I interferon (IFN-β) production (↑3.2-fold compared to SDT alone) (84, 85). This IFN microenvironment upregulates tumor cell PD-L1 expression via JAK1/STAT1 signaling. Meanwhile, ICIs disrupt PD-1/PD-L1 dimerization, block SHP-1/2 recruitment, and restore PI3K-AKT and MAPK signaling in CD8^+^ T cells, resulting in a 2.8-fold increase in granzyme B and perforin release (90). Temporal coordination is critical: SDT-induced IFN-β peaks at 24–48 h, coinciding with T cell activation; ICI-induced de-suppression sustains effector function, achieving >85% tumor inhibition in preclinical models (64). In solid tumors such as breast cancer and colorectal cancer models, SDT-ICI nanocomposites significantly improve tumor inhibition rates, suppress distant metastasis, and induce systemic immune memory, reducing the risk of recurrence (90, 91).

Although the combination of SDT and ICIs shows great promise with optimized nanomaterials, it still faces challenges, such as limited ROS production efficiency due to the quantum yield of Sonosensitizers and insufficient oxygen supply, as well as systemic toxicity of ICIs that may lead to immune-related adverse events. To overcome these challenges, researchers have developed multifunctional nanoscale systems, such as integrating catalytic enzyme mimics (e.g., MnO_2_ nanoparticles) to alleviate tumor hypoxia or combining photothermal effects to enhance vascular permeability and promote drug delivery. Preclinical experiments have confirmed that in hepatocellular carcinoma models, anti-PD-L1-loaded sonosensitized nanobubbles combined with SDT not only amplify local tumor ablation but also activate anti-tumor immunity, inhibit tumor growth, and improve survival (92). Additionally, in refractory tumors such as gliomas, the nano-mediated SDT-ICI strategy achieved effective abscopal effects through blood-brain barrier penetration design, meaning that treating the primary tumor inhibited untreated distant lesions (93). In the future, with advancements in nanotechnology, such as smart responsive carriers and personalized sound-sensitive agent design, this combined therapy is expected to enter clinical trials, driving a paradigm shift in precision tumor immunotherapy. However, long-term safety and resistance mechanisms require further investigation.Based on the above discussion, we have compiled the following tumor classification comparison table (**Table 2**).

**Table 2:** Various types of tumors and their corresponding SDT therapies.

Tumor type	Nanoparticle design name	Tumor model	Treatment strategy	Key findings	References
breast cancer	IMP@CM-PEP20 NPs	4T1 breast tumor mouse model	SDT synergistic CD47 blockade immunotherapy	IMP@CM-PEP20 NPs enhance tumor-targeted delivery and macrophage phagocytosis by inducing ferroptosis through MnO2-mediated glutathione depletion. IR780 generates singlet oxygen under ultrasound to amplify ferroptosis and M2-to-M1 macrophage reprogramming. Ferroptosis-mediated ICD stimulates dendritic cell antigen presentation, activates cytotoxic T cell immunity, and establishes persistent immune memory.	(13)
pancreatic cancer	SPNs	Panc02 cell line *in situ* model	SDT synergistic immunotherapy with Toll-like receptor 7/8 agonists and IDO inhibitors	SPNs improve tumor penetration and remodel the tumor microenvironment by degrading hyaluronic acid. Under sound activation, they generate singlet oxygen for SDT, induce ICD, and trigger the controlled release of R848 and NLG919, activating dendritic cells and inhibiting IDO, resulting in a powerful anti-tumor immune response that almost completely suppresses tumor growth and inhibits metastasis.	(94)
glioblastoma	AMNP@J + C	GL261 cell line *in situ* model	SDT synergistic PD-L1 checkpoint blockade immunotherapy	AMNP@J + C achieves co-delivery of Ce6 and JQ1, producing synergistic antitumor effects *in vitro* and *in vivo* and improving survival in GBM mice. SDT generates ROS to kill tumor cells and induce ICD, while JQ1 inhibits tumor proliferation and PD-L1 expression, enhancing the antitumor immune response.	(95)
colorectal cancer	QD/POM1@NP@M	Subcutaneous model of CT26 cell line	SDT synergistic PD-L1 checkpoint blockade immunotherapy	QD/POM1@NP@M encapsulates Ag2S quantum dots and the CD39 inhibitor POM1, with homologous tumor cell membrane coating enhancing targeting. SDT induces ICD to release ATP, triggering an immune response. POM1 inhibits CD39 to reduce adenosine accumulation, improve the immunosuppressive microenvironment, promote anti-tumor immune cell infiltration, and transform the tumor from “cold” to “hot.” Combining with α-PDL1 enhances systemic anti-tumor immunity and promotes long-term immune memory.	(96)
bladder cancer	SPCP/CCP@Bay	MB49 cell line model	SDT synergistic PD-1 monoclonal antibody immunotherapy	SPCP/CCP@Bay combines SDT and disulfidptosis to enhance ICD and improve the immunosuppressive tumor microenvironment. Under ultrasound guidance, Bay-876 is released to disrupt redox balance, induce disulfidptosis, and synergistically inhibit tumor growth with α-PD-1, transforming “cold tumors” into “hot tumors” for advanced cancer immunotherapy.	(97)
prostate cancer	Ce6@aPD-L1 NBs	PC-3 cell line model	SDT synergistic aPD-L1 immune checkpoint blockade therapy	Ce6@aPD-L1 NBs promote tumor-targeted delivery, activating antitumor effects through direct action of SDT and reactivation of the immune system. SDT induces ROS generation and ICD, while aPD-L1 blocks checkpoints to enhance immune responses and inhibit prostate cancer growth.	(98)
ovarian cancer	FSC nanorobot collectives	SKOV3 cell line model	SDT synergistic magnetic drive enhances immune-targeted therapy	FSC nanorobots collectively enhance targeting through magnetic drive, induce ICD through SDT, promote immune cell recruitment and activation, significantly inhibit tumors, and prolong survival, demonstrating potential for clinical translation.	(99)
lung cancer	Polymeric nanoplatform	A549 cell line model	SDT synergistic chemotherapy and immunotherapy	Polymer nanoplates loaded with sound-sensitive agents and chemotherapy drugs enhance ROS production under ultrasound, induce ICD, boost anti-tumor immune response, and improve the treatment effect of deep lung cancer.	(100)

## Discussion

The technologies reviewed herein demonstrate significant promise in preclinical studies for sonodynamic therapy (SDT), with potential clinical utility, though their translation faces specific obstacles beyond those in traditional therapies. Key issues include optimizing nanodelivery systems to enhance tumor targeting while minimizing off-target effects, as fabrication demands specialized infrastructure that escalates costs and hinders scalability (101). Safety concerns, such as nanomaterials inducing long-term toxicity or inadequate biocompatibility, require thorough preclinical assessment (102). Regulatory hurdles delay approval of novel combinations, necessitating exhaustive data from studies (103). Nevertheless, these challenges underscore the need for innovation to move SDT toward clinical reality, building on its unique ability to induce immunogenic cell death (ICD) and reprogram the tumor microenvironment (TME) (16).

Emerging nanotechnology advancements provide tailored solutions for SDT, focusing on precise delivery and synergy with immunotherapy. Biodegradable nanocarriers, such as poly(lactic-co-glycolic acid) (PLGA)-based systems, enable ultrasound-responsive degradation for sustained sonosensitizer release, achieving precise SDT while avoiding chronic residue toxicity. CRISPR-based gene editing targets tumor-related mutations, such as drug-resistant genes, enhancing ROS sensitivity and SDT efficacy by up to 30% in preclinical models, though efficient delivery and off-target effects need addressing (52). AI machine learning algorithms predict optimal nanoparticle formulations and personalize SDT regimens, simulating ultrasound propagation to optimize loading rates and improve efficacy by 20–40%, with applications like deep learning reducing experimental iteration cycles by 50% (104). Overall, integrating these technologies positions SDT nanostructures for efficient, minimally invasive cancer therapies, particularly in overcoming TME immunosuppression.

## Summary and outlook

SDT combines nanodelivery systems with ultrasound therapy, significantly enhancing therapeutic efficacy and precision. This therapy utilizes sonosensitizers, which, when activated by low-intensity ultrasound, generate reactive oxygen species (ROS). These substances trigger tumor cell death, disrupt cellular structures, and initiate a therapeutic cascade. Advances in sonosensitizer development, from first-generation natural compounds to third-generation synthetic molecules and then to nanomaterials, have enhanced therapeutic specificity and reduced side effects. SDT exhibits potent antitumor activity against a variety of malignancies, including melanoma, colorectal adenocarcinoma, lung cancer, and head and neck cancers, primarily through ROS-mediated cytotoxicity and selective tumor targeting. Sonosensitizers convert ultrasound energy into ROS, causing oxidative damage to tumor cells and disrupting their vascular networks. Ideal sonosensitizers require high water solubility, excellent biocompatibility, efficient ROS conversion, and precise tumor targeting. This therapy holds great potential for treating malignancies such as breast cancer, neuroblastoma, and prostate cancer, modulating the tumor microenvironment and enhancing drug penetration. Nanodelivery systems have revolutionized the field of sonotherapy by improving the solubility, stability, and tumor targeting of sonosensitizers. Combining SDT with chemotherapy can produce a synergistic effect, significantly enhancing overall therapeutic efficacy. The core mechanism of this synergistic effect lies in the reactive oxygen species (ROS) generated by SDT, which not only directly kill tumor cells but also trigger a therapeutic cascade by disrupting tumor structure. In short, the advantages of combining SDT with nanodelivery technologies lie in their minimal invasiveness and high selectivity, enabling personalized treatment plans tailored to tumor heterogeneity and advancing cancer treatment.

Despite the progress made in sonodynamic therapy, two major factors remain significant challenges: the limitations of ultrasound technology and the inherent imperfections of nanoparticle formulations. 1. Ultrasound-related factors: Ultrasound penetration depth limits therapeutic efficacy, especially in deep-seated tumors, where acoustic attenuation results in approximately 30% energy loss—for example, in bone tumor treatment—underscoring the need for high-intensity focused ultrasound (HIFU) systems to maintain efficacy. Overcoming these limitations requires breakthroughs in ultrasound sources and imaging technologies. 2. Nanomaterial-related factors: (i) Nanoparticle size, composition, and surface properties decisively influence their ***in vivo*** behavior, but these properties dynamically change after administration due to biological interactions, complicating performance evaluation. (ii) The reticuloendothelial system (RES) rapidly clears nanoparticles, reducing tumor bioavailability and potentially inducing hepato-splenic toxicity. Polyethylene glycol (PEG) modification can only provide limited mitigation of this problem. (iii) Tumor heterogeneity and microenvironmental barriers hinder both ligand-mediated active targeting and passive accumulation via the enhanced permeability retention (EPR) effect, thereby hindering reliable drug delivery. (iv) Unresolved safety concerns of nanomaterials include insufficient biodegradability, genotoxicity, metal-induced neurotoxicity, and chronic inflammatory responses, all of which may reduce therapeutic efficacy. Furthermore, although SDT is typically a minimally invasive procedure, excessive generation of reactive oxygen species (ROS) can lead to off-target toxicity and damage surrounding healthy tissues. Improving activation control and targeting precision remains a research priority. The development of novel nanomaterials through innovative material design and surface modification requires validation of their enhanced biocompatibility, targeting specificity, and ROS generation efficiency in the complex tumor microenvironment. Comprehensive safety and efficacy evaluations are essential prerequisites for integrating nanodelivery systems and SDT into clinical practice.

Future applications of nano-SDT in tumor immunotherapy will trend toward multimodal, precision-engineered platforms. By optimizing nanodelivery systems and sonosensitizer design, this technology can enhance immunogenic cell death (ICD) effects and advance clinical application. However, several challenges remain before achieving large-scale clinical translation, including formulation issues (such as biocompatibility and minimizing toxicity), scalable manufacturing (such as reproducibility and cost-effectiveness), biological and clinical translation barriers (such as RES clearance and TME restrictions), and therapeutic efficacy limitations (such as ROS generation efficiency and tumor heterogeneity). These challenges highlight current bottlenecks compared to recent studies. For example, the MnO_2_ system enhances ROS production by alleviating hypoxia, while other systems require further optimization of targeting depth. These bottlenecks currently hinder its widespread application.

To bridge the gap between laboratory research and clinical practice and achieve the best therapeutic effect of SDT, innovations are urgently needed in nanoparticle design (focusing on enhancing sonosensitizer delivery, ROS generation, tumor targeting, and synergy with immunotherapy or other modalities), targeting strategies (tumor microenvironment-responsive carriers), and toxicity management (compatible materials). Current research is overcoming these bottlenecks through breakthrough nanoparticle design and targeting strategies. For example, the biomimetic nano-erythrocyte platform targets tumor-associated macrophages, which can reconstruct the immunosuppressive tumor microenvironment (TME) and significantly improve the effect of chemotherapy and immunotherapy (105). Bimetallic nanoparticles, as cascade sensitization amplifiers, provide new ideas for low-dose radioimmunotherapy and open up new paths for TME-responsive design (106). We believe that new treatment options based on SDT will gradually mature, and the role of ultrasound therapy in cancer diagnosis and treatment will become increasingly prominent. This opens up new paths for precision medicine and benefits more patients. In particular, in clinical applications, the non-invasive nature of SDT makes it an ideal choice for people with surgical contraindications (such as elderly patients or patients with metastatic cancer). When used in combination with chemotherapy, it can reduce chemotherapy doses by 25% and minimize side effects. Optimizing nanoparticle delivery systems (such as liposome-encapsulated sonosensitizers) can double reactive oxygen species (ROS) production, addressing insufficient production. Focused ultrasound technology significantly reduces attenuation, lowering the risk of damage to less than 4%. Integrating multimodal nanoplatforms (such as SDT-chemotherapy combination systems) has improved local efficacy in carcinoma *in situ* by 40%. To address challenges in clinical translation: automated synthesis systems precisely control nanoparticle size (ranging from 15–40 nanometers), ensuring *in vivo* distribution uniformity below 8 nanometers. Reticuloendothelial system (RES) clearance reaches 70%, while PEGylation only reduces it by 15%. A novel erythrocyte membrane-mimicking coating extends circulation time by 1.5-fold. Artificial intelligence predictive models optimize EPR effect variations, enhance ligand density, and increase drug accumulation by 35%. Furthermore, non-metallic organic sonosensitizers can reduce the risk of DNA damage to less than 1%, and their blood-brain barrier design effectively avoids neurotoxicity. Standardized operating procedures eliminate batch variability, and large-scale production reduces costs to $1,500 per dose. These technological breakthroughs have accelerated the clinical application of SDT and promoted the development of cancer treatment towards personalized and non-invasive directions.

## Conclusions

In summary, nanoparticle-mediated SDT represents a promising frontier in tumor immunotherapy by inducing ICD and reprogramming the tumor microenvironment, overcoming limitations of traditional treatments and enhancing the efficacy of immunotherapy and chemotherapy. Despite challenges in ROS production, clinical translation, and nanocarrier optimization, recent advancements in multifunctional nanoplatforms, biodegradable carriers, and AI-driven designs offer innovative solutions, paving the way for precise, low-toxicity, and personalized cancer therapies with significant clinical potential.
